# Identification of Reference Genes for Quantitative Expression Analysis of MicroRNAs and mRNAs in Barley under Various Stress Conditions

**DOI:** 10.1371/journal.pone.0118503

**Published:** 2015-03-20

**Authors:** Jannatul Ferdous, Yuan Li, Nicolas Reid, Peter Langridge, Bu-Jun Shi, Penny J. Tricker

**Affiliations:** Australian Centre for Plant Functional Genomics, University of Adelaide, Hartley Grove, Urrbrae, South Australia 5064, Australia; University of Münster, GERMANY

## Abstract

For accurate and reliable gene expression analysis using quantitative real-time reverse transcription PCR (qPCR), the selection of appropriate reference genes as an internal control for normalization is crucial. We hypothesized that non-coding, small nucleolar RNAs (snoRNAs) would be stably expressed in different barley varieties and under different experimental treatments, in different tissues and at different developmental stages of plant growth and therefore might prove to be suitable reference genes for expression analysis of both microRNAs (miRNAs) and mRNAs. In this study, we examined the expression stability of ten candidate reference genes in six barley genotypes under five experimental stresses, drought, fungal infection, boron toxicity, nutrient deficiency and salinity. We compared four commonly used housekeeping genes; Actin (*ACT*), alpha-Tubulin (*α-TUB*), Glycolytic glyceraldehyde-3-phosphate dehydrogenase (*GAPDH*), ADP-ribosylation factor 1-like protein (*ADP*), four snoRNAs; (U18, U61, snoR14 and snoR23) and two microRNAs (miR168, miR159) as candidate reference genes. We found that *ADP*, snoR14 and snoR23 were ranked as the best of these candidates across diverse samples. For accurate and reliable gene expression analysis using quantitative real-time reverse transcription PCR (qPCR), the selection of appropriate reference genes as an internal control for normalization is crucial. We hypothesized that non-coding, small nucleolar RNAs (snoRNAs) would be stably expressed in different barley varieties and under different experimental treatments, in different tissues and at different developmental stages of plant growth and therefore might prove to be suitable reference genes for expression analysis of both microRNAs (miRNAs) and mRNAs. In this study, we examined the expression stability of ten candidate reference genes in six barley genotypes under five experimental stresses, drought, fungal infection, boron toxicity, nutrient deficiency and salinity. We compared four commonly used housekeeping genes; Actin (*ACT*), alpha-Tubulin (*α-TUB*), Glycolytic glyceraldehyde-3-phosphate dehydrogenase (*GAPDH*), ADP-ribosylation factor 1-like protein (*ADP*), four snoRNAs; (U18, U61, snoR14 and snoR23) and two microRNAs (miR168, miR159) as candidate reference genes. We found that *ADP*, snoR14 and snoR23 were ranked as the best of these candidates across diverse samples. Additionally, we found that miR168 was a suitable reference gene for expression analysis in barley. Finally, we validated the performance of our stable and unstable candidate reference genes for both mRNA and miRNA qPCR data normalization under different stress conditions and demonstrated the superiority of the stable candidates. Our data demonstrate the suitability of barley snoRNAs and miRNAs as potential reference genes for miRNA and mRNA qPCR data normalization under different stress treatments.

## Introduction

The study of gene expression has become increasingly widespread in numerous organisms. Real time quantitative (reverse transcription) polymerase chain reaction (qPCR) is a commonly used technique due to its high sensitivity, accuracy and reproducibility. Among several quantification strategies, a commonly used method is relative quantification where data are normalized to an internal control gene [1]. The internal control gene is called a reference or house-keeping gene (HKG) and, while subjected to the same experimental factors during sampling and cDNA preparation as the gene(s) of interest, is not differentially expressed, providing a constant for relative quantification of differences in expression of the gene(s) of interest. Hence, the selection of appropriate reference genes is important for obtaining valid results and proper interpretation from the analysis [2]. The most frequently used HKGs in plant qPCR analysis are protein-coding genes, such as Actin (*ACT*), alpha-Tubulin (*α-TUB*), Cyclophilin, Glyceraldehyde-3-phosphate dehydrogenase (*GAPDH*), ADP-ribosylation factor 1-like protein (*ADP*) and ribosomal RNAs [3–9]. However, several studies have shown that the expression of these genes varies considerably in different cells and tissues under different experimental conditions and, in these comparisons, they become unsuitable for qPCR data normalization [9–16].

Until two decades ago it was believed that the DNA between protein-coding genes was nothing more than junk DNA [17]. This idea was challenged by the discovery of small regulatory RNAs in eukaryotes. MicroRNAs (miRNAs) are a class of small RNAs, approximately 18–24 nucleotides (nt) long, non-coding and single-stranded molecules. miRNAs play pivotal roles in cellular homeostasis by the alteration of gene expression under stress conditions [18, 19]. Recent advances in high-throughput sequencing and bioinformatics analyses have enhanced the discovery of miRNAs in different plant species [20–23]. However, the regulatory function of miRNAs is just beginning to be understood [24–26]. miRNAs negatively regulate their target messenger RNAs (mRNAs) and the regulation is subject to various levels of control [18]. To determine the function of miRNAs, the expression levels of miRNAs and their target mRNA *in vivo* must be precisely compared. To investigate the differential expression of miRNAs, sequencing and computational analyses require the experimental validation of expression profiles. However, miRNA experimental validation is difficult because of their short length (∼ 18–24 nt) and lack of common sequence features (e.g. polyA) [27].

Barley (*Hordeum vulgare*) is one of the most important cereal crops cultivated in the world and, with a large amount of genetic and genomic data available, including its full genome sequence [28], is a model for cereal genomics studies. Although several reports have suggested suitable reference genes for RNA quantification in cereals, all show some variation in expression amongst plant tissues, species and experimental conditions so that there is no known reference genes suitable for all experiments [29–32]. Additionally, inadequate information is available on suitable reference genes for miRNA-qPCR. Studies of miRNA expression using qPCR often provide limited information on the stability of reference genes, but, where investigated, lower expression stability of some commonly used HKGs has been found in miRNA-qPCR experiments [2, 33, 34]. The use of miRNAs [1, 27, 35–37] and small nucleolar RNAs (snoRNAs) [38, 39] as reference genes has been proposed for better normalization in miRNA-qPCR expression analysis in both plants and animals. However, barley miRNAs and snoRNAs have never been assessed for their expression stability as reference genes under biotic and abiotic stresses.

The present work was designed to identify and evaluate suitable reference genes in barley under drought, salinity, boron toxicity, low nutrient stress and fungal infection. The selected internal controls were used for miRNA and mRNA expression profiling in barley to validate the performance of stable reference genes under drought stress treatment. Candidate reference genes were examined and compared with commonly used HKGs and ranked with three statistical algorithms. We found that the expression of four commonly used HKGs varied with experimental conditions. By contrast, snoRNAs and miRNAs were suitable internal controls for miRNA and mRNA-qPCR expression analysis in barley.

## Materials and Methods

### Plants and Growth Environments

#### Drought treatment

*H*. *vulgare* cvs. ‘Fleet’ and ‘Commander’ were grown in a glasshouse at 22–23°C day (d)/16°C night temperatures, with a day length of 12 hours (h). Plants were grown in pots with coco-peat soil. 30 individual plants of each genotype were grown. Drought treatment was applied as follows: plants were initially well-watered, then water withheld until visible wilting. Samples were collected after 21 d, after flag leaf emergence and 10 d after anthesis. The sampled materials corresponded to three developmental stages: tillering, booting and grain filling. Consistently well-watered plants were sampled as control plants. Leaf and root materials were sampled at tillering and booting stages and grains were harvested at the grain filling stage, 18 d post-anthesis.

#### Boron treatment

*H*. *vulgare* cv. ‘Clipper’ plants were grown hydroponically for 21 d in sufficient and excessive concentrations of boron with temperatures and day length as above. Seedlings were grown in a nutrient solution containing 5 mM NH_4_NO_3_, 5 mM KNO_3_, 2 mM Ca(NO_3_)_2_, 2 mM MgSO_4_, 100 μM KH_2_PO_4_, 50 μM NaFe(III) EDTA, 50 μM B(OH)_3_, 5 μM MnCl_2_, 10 μM ZnSO_4_, 0.5 μM CuSO_4_ and 0.1 μM Na_2_MoO_3_. After 7 d, half of the seedlings were transferred to a nutrient solution with an additional 3 mM H_3_BO_3_. Leaves were harvested after 14 d.

#### Fungal infection

*H*. *vulgare* cv. ‘Sloop’ seeds were sown in pots with coco-peat soil in a growth chamber at 18°C with a 14/10 h day/night light period and 60±10% relative humidity (RH). After three weeks, three individual plants were infected with *Rhynchosporium commune*. After inoculation the seedlings were maintained in darkness for 24 h at 100% RH, then returned to the 14/10 h day/night light period with 80±10% RH until the leaf samples were harvested, 14 d after inoculation. Same age uninfected plant leaves from three individual plants were harvested as the control group.

#### Salt treatment

*H*. *vulgare* cv. ‘WI4330’ seedlings were grown hydroponically as described for the boron treatment. At the appearance of the third leaf (approx. 10 d), half of the seedlings were subjected to the treatment by transferring them to a nutrient solution with an additional 50 mM NaCl. NaCl was added to the nutrient solution twice daily in increments of 50 mM, to a final concentration of 250 mM. Roots were harvested for RNA extraction when the third leaf was fully expanded approximately after a further 12 d.

#### Nitrate treatment

*H*. *vulgare* cv. ‘Golden Promise’ seedlings were grown in a fully-supported hydroponics set-up [40] in a glasshouse with a temperatures ranging between 19–23°C. Sufficient and low nitrate treatments were achieved by supplying 5 mM and 0.5 mM nitrate to the nutrient solution, respectively. The nutrient solution contained (in mM): 2.0 MgSO_4_.7H_2_O, 0.1 KH_2_PO_4_, 0.5 Na_2_Si_3_0_7_, 0.05 NaFe(III)EDTA, 0.05 H_3_BO_3_, 0.005 MnCl_2_.4H_2_O, 0.01 ZnSO4.7H_2_O, 0.0005 CuSO4.7H_2_O and 0.0001 Na_2_MoO_4_.2H_2_O with 2 KNO_3_ and 1.5 Ca(NO_3_)_2_.4H_2_O in the sufficient NO_3_^−^ treatment (5 mM NO_3_^−^) and 0.25 KNO_3_ and 0.125 Ca(NO_3_)_2_.4H_2_O in the low NO_3_^-^ treatment (0.5 mM NO_3_^−^). In order to maintain similar K^+^ and Ca^2+^ levels to the sufficient NO_3_^−^ treatment, the low NO_3_^−^ treatment also comprised (in mM): 0.875 K_2_SO_4_ and 1.375 CaCl_2_.2H_2_O. The treatments were established at the start of the experiment and the nutrient solution was replaced every 10 d to ensure that nutrients were not depleted. Following 21 d of sufficient and low nitrate treatment, the 2nd leaf blade was collected.

All harvested materials were immediately frozen in liquid N_2_ and stored at −80°C.

### Candidate Reference Gene Selection and Primer Design

Four common HKGs (*ACT*, *α-TUB*, *GAPDH* and *ADP*), four snoRNAs (U18, U61, snoR14 and snoR23) and two miRNAs (miR168 and miR159) were selected. The sequences of *Hvu-Actin* (AY145451.1: http://www.ncbi.nlm.nih.gov/nuccore/AY145451), *Hvu-Tubulin* (U40042.1: http://www.ncbi.nlm.nih.gov/nuccore/U40042.1), *Hvu-GAPDH* (X60343.1: http://www.ncbi.nlm.nih.gov/nuccore/X60343.1) and *Hvu-ADP* (AJ508228.2: http://www.ncbi.nlm.nih.gov/nuccore/AJ508228.2) were downloaded from the NCBI Genbank while the sequences of all barley snoRNAs were downloaded from the plant snoRNA database (http://bioinf.scri.sari.ac.uk/cgi-bin/plant_snorna/home). The sequences of miR168 (accession number MIMAT0018216) and miR159 (accession number MIMAT0018210) were obtained from miRBase (http://www.mirbase.org/index.shtml). The selection of these two miRNAs was based on our previous miRNA expression studies in drought-treated *H*. *vulgare* cv. ‘Golden Promise’, where these two miRNAs were relatively invariant across drought treated and well-watered samples (data not shown).

Primers for *ACT*, *α-TUB*, *GAPDH*, *ADP* and snoRNAs were designed using AlleleID software (Premier Biosoft International, Palo Alto, CA, USA) considering amplicon sizes in the range of 60–80 bases. To eliminate the possibility of an inhibiting effect of the RNA secondary structure during cDNA synthesis, the primers for snoRNAs were designed in their loop regions [41]. miRNA specific stem-loop RT primers and appropriate forward and reverse primers for individual miRNA were designed following the previously described method [42, 43]. A minimum of three primer pairs were designed and tested for each gene. Primer pairs for 10 genes were selected on the basis of their amplification efficiency and specificity and are listed in Table 1. All primers used in this study were synthesized by Integrated DNA Technologies (IDT, Coralville, IA, USA).

**Table 1:** Ten candidate reference genes and primer sequences used for their qPCR.

Candidate reference genes	Accession number	Annotation	Primer (5′–3′)	Amplicon (bp)	PCR efficiency	Regression coefficient (R^2^)
Hvu-U61	-	Small nucleolar RNA	Fw GAGGAAACGAAACCTGTGC	65	93.94	0.999
			Rev ACTTCTTAGAGGGTTGTGTTAC			
Hvu-U18	-	Small nucleolar RNA	Fw GTGATGAAGAAAAGTTGGTC	67	89.25	0.999
			Rev AGAAGTTTATTAAGGATGGTTATC			
Hvu-snoR14	-	Small nucleolar RNA	Fw GATGTTTATGTATGATAGTCTGTC	67	95.55	0.999
			Rev GTCGGGATGTATGCGTGTC			
Hvu-snoR23	-	Small nucleolar RNA	Fw TCGGCAGTGGTGTCATC	64	98.31	0.999
			Rev CTCAGTGGAAAGAGAAGTCG			
Hvu-ADP	AJ508228.2	ADP-ribosylation factor 1-like protein	Fw GCTCTCCAACAACATTGCCAAC	77	100.79	0.999
			Rev GAGACATCCAGCATCATTCATTCC			
Hvu-α-TUB	U40042.1	Tubulin alpha-2 chain	Fw GTCCACCCACTCCCTCCTTG	78	106.49	0.999
			Rev CGGCGGCAGATGTCATAGATG			
Hvu-ACT	AY145451.1	Actin	Fw CCACGAGACGACCTACAAC	80	102.36	0.999
			Rev CACTGAGCACGATGTTTCC			
Hvu-GAPDH	X60343.1	Glycolytic glyceraldehyde-3-phosphate dehydrogenase	Fw GCCAAGACCCAGTAGAGC	78	92.12	0.999
			Rev CACATTTATTCCCATAGACAAAGG			
Hvu-miR168	MIMAT0018216	microRNA	Fw CTCACGTCGCTTGGTGCAGAT	60	107.31	0.997
			Rev GAGCTGGGTCCGAGGT			
			Stem-loop RT primer GTCGTATCCAGAGCTGGGTCCGAGGTATTCGCTCTGGATACGACGTCCCG			
Hvu-miR159	MIMAT0018210	microRNA	Fw CGTGGGTTTGGATTGAAGGGA	61	107.21	0.999
			Rev GTGCAGGGTCCGAGGT			
			Stem-loop RT primer GTCGTATCCAGTGCAGGGTCCGAGGTATTCGCACTGGATACGACCAGAGC			

Specific qPCR amplification for all candidate internal controls was confirmed by a single, distinct melt peak in melt curve analysis (S1 Fig.) and a single band of desired size in 3% agarose gel electrophoresis (S2 Fig.). Further confirmation of these qPCR amplified products was done by sequencing using the respective reverse primers each containing M13 reverse primer sequence and a spacer (S1 Table), and all of these qPCR products showed correct sequences.

### qPCR Analysis

Total RNA was extracted from each of the samples using TRIzol reagent (Invitrogen, Carlsbad, CA, USA) according to the manufacturer’s instructions, except for grain RNA extraction, which included additional 2% (w/v) polyvinylpyrrolidone (PVP-40) (Sigma). From each of the five experiments RNA was isolated from a minimum of three biological replicates from each of the treated and control conditions.

The isolated RNA samples were treated with DNA-free reagents (Ambion, Life Technologies, Grand Island, NY, USA) twice according to the manufacturer’s instruction in order to completely remove genomic DNA (gDNA). A polymerase chain reaction (PCR) was carried out to ensure no gDNA contamination in the DNAse-treated RNA samples (S3 Fig.). The concentration and integrity of the DNA-free RNA was determined by Agilent-2100 Bioanalyzer using RNA 6000 NanoChips (Agilent Technologies, Santa Clara, CA, USA). RNA samples with RNA integrity numbers (RIN) ≥ 5 were used for cDNA synthesis. cDNAs were synthesized in a final volume of 40 μl. In each 40 μl reaction, the reaction mixture contained 2 μg RNA, 2 μl (10 mM) dNTP mix and a cocktail of 2 μl (1 μM) appropriate stem loop primer, 2 μl (10 mM) appropriate snoRNA specific reverse primers and 2 μl 50 μM Oligo (dT)_20_ (Life Technologies, Carlsbad, CA, USA) (Table 1). cDNA synthesis was carried out using SuperScript III RT (Life Technologies, Carlsbad, CA, USA). All investigated samples were transcribed by the pulsed RT method recommended for stem-loop primers [43]. In brief, the samples were incubated at 16°C for 30 minutes (min) followed by pulsed RT of 60 cycles at 30°C for 30 seconds (s), 42°C for 30 s and 50°C for 1 s. Finally, the reaction was terminated by incubating the samples at 85°C for 5 min.

To provide a template for the standard curve, four (three technical replicates and one no template control) 20-μL PCR reaction mixtures were combined and purified using illustra GFX PCR DNA and Gel Band Purification Kit (GE Healthcare). The purified product was then quantified using the QUBIT fluorometer and the Qubit dsDNA HS assay kit (Life Technologies) according to the manufacturer’s instructions. An aliquot of this solution was diluted to produce a stock solution containing10^9^ copies of the PCR product per μL. A dilution series covering six orders of magnitude was prepared from the 10^9^ stock solution to produce solutions covering 10^7^ to 10^1^ copies per μL to derive a standard curve (S4 Fig.) for each assay. Reactions were prepared in triplicates and a no template control.

qPCR assays were assembled using the fluid-handling robotic platform CAS-1200 (QIAgility, Qiagen, Valencia, CA, USA). The cDNA samples were diluted 20 times in sterile milli Q water and the diluted cDNA solution was used in the qPCR reaction. Each assay contained: 2 μl of cDNA solution (or water as a no template control), 5 μl of KAPA Sybr Fast qPCR Universal Readymix (Geneworks, Adelaide, Australia), 1.2 μl of each of the forward and reverse primers at 4 μM and 0.6 μl water to make up the total reaction volume of 10 μl. Amplifications were performed in an RG 6000 Rotor-Gene Real-Time Thermal Cycler (Qiagen, Valencia, CA, USA) with 2 min at 95°C followed by 50 cycles of 1 s at 95°C, 1 s at 60°C, 25 s at 72°C, and fluorescent acquisition at 72°C, followed by melt curve analysis of temperature increasing from 60°C to 95°C with fluorescence readings acquired at 0.5°C increments (S1 Fig.).

### Gene Expression Stability Ranking of Candidate Reference Genes

The stability of candidate reference genes’ expression was analysed using three software packages, geNorm (version 3.5) ([3] http://www.biogazelle.com/genormplus/website), Normfinder (version 0.953) ([44] http://www.mdl.dk/publicationsnormfinder.htm webcite) and BestKeeper (version 1) ([4] http://genequantification.com/bestkeeper.html). Raw expression values of candidate reference genes obtained from the qPCR under five experimental treatments were analysed by geNorm and NormFinder according to the instruction manual. geNorm was used to make pairwise comparisons and exclude values stepwise until the most stable pair of genes remained [3]. NormFinder was used to examine the expression stability of each candidate independently [44]. Both software packages were used to calculate the average expression stability (M) of candidate reference genes, with the highest M value indicating the least stable candidate reference genes and the lowest M value the most stable [3] and to rank their performance accordingly. BestKeeper analysis was used to calculate the geometric mean of the Cycle threshold (Ct) values of the candidate reference genes [4] and estimate gene expression stability based on the standard deviation of the Ct value, coefficient of correlation (r) and percentage covariance, to rank the candidates from the most to least stably expressed.

### Determination of the Optimal Number of Reference Genes

To determine the optimal number of reference genes, the pairwise variation, V, for all datasets was estimated using geNorm (as before). V was calculated between two sequential normalization factors NFn and NFn+1 to determine the optimal number of reference genes [3]. If V was below 0.15, the addition of a third gene did not result in a noticeable improvement for the normalization. In particular, if Vn/n+1 was less than 0.15, using n+1 reference genes did not significantly improve the normalization [3].

### Validation of Candidate Reference Genes’ Utility

To validate the reliability of putatively more stable candidate reference genes, the relative expression levels of an mRNA (*Superoxide dismutase*) and an miRNA (miR5048) (S2 Table) were also quantified for all the samples described above by qPCR (as before). Melt curves and standard curves in qPCR for S*uperoxide dismutase* (AK363344.1) and miR5048 (MIMAT0020544) are given in S5 Fig. and S6 Fig., respectively. Expression of miR5048 was compared with previously described deep sequencing data [45].

## Results

### Gene Expression Stability and Ranking of Candidate Reference Genes by the geNorm analysis

**Fig 1:**
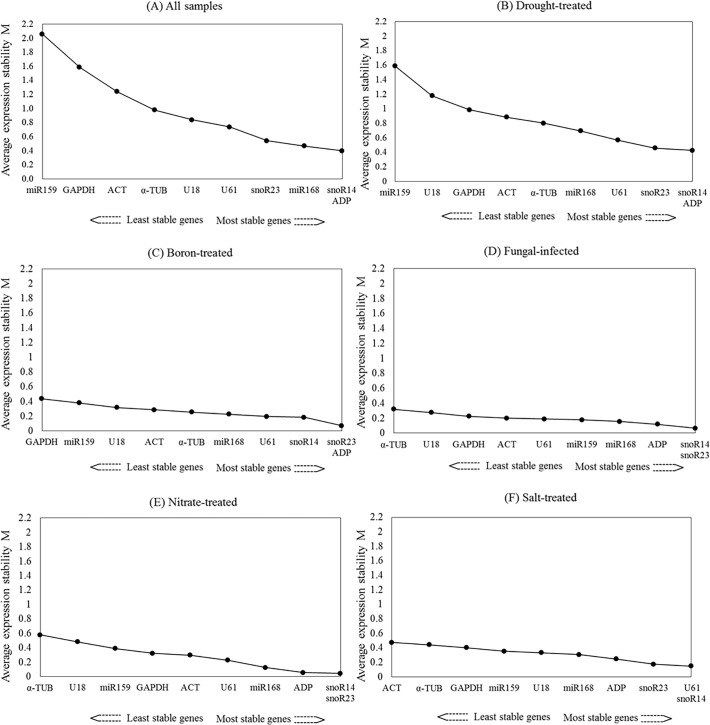
geNorm analysis of average expression stability values and ranking of ten candidate reference genes based on pairwise comparison. Genes on the x-axis in order of increasing stability (y-axis M value) for (A) all samples, (B) drought-treated samples, (C) boron-treated samples, (D) fungal-infected samples, (E) salt-treated samples and (F) nitrate-treated samples. Under individual treatment condition, reference genes’ expression stability was obtained comparing to the untreated (control) condition.

Average expression stabilities (M) for candidate reference genes using pairwise comparison are shown in Fig. 1. snoR14 and *ADP* had the lowest M value (0.399), that is the most stable expression amongst all 10 candidate reference genes across all tissue samples. In contrast, miR159 had the highest M value (2.056) that is the least stable expression across all samples (Fig. 1A). Under drought stress, the most stable reference genes were *ADP* and snoR14, and the least stable was again miR159 (Fig. 1B). Under boron treatment the most stable reference genes were snoR23 and *ADP*, and the least stable reference gene was *GAPDH* (Fig. 1C). Under both fungal infection (Fig. 1D) and nitrate treatment (Fig. 1E) snoR14 and snoR23 were the most stable candidates. *α-TUB* was the least stable gene under both fungal infection (Fig. 1D) and nitrate treatment (Fig. 1E). Under salt treatment, U61 and snoR14 were the most stable candidates while *ACT* was the least stable candidate (Fig. 1F).

### Gene Expression Stability and Ranking of Candidate Reference Genes by the Norm Finder analysis

The most stable reference gene identified by NormFinder across all samples was snoR14. *ADP* and snoR23 were ranked second and third, respectively. snoR14, *ADP* and snoR23 also performed well under boron, fungal and nitrate stresses (Table 2). miR159 was the least stable (M >1) when used as a universal reference gene across all experimental samples. Though the M value was < 1, *α-TUB* was the most unstable candidate in fungal and nitrate treatment conditions (Table 2). *ACT* and *GAPDH* were the least stable candidates in salinity and boron treatments respectively. These results were broadly consistent with the pairwise analysis using the geNorm algorithm (Fig. 1).

**Table 2:** Expression stability ranking of candidate reference genes as calculated by NormFinder and BestKeeper.

		Experimental conditions					
	Rank	All samples	Drought	Boron	Fungus	Salt	Nitrate
NormFinder (Stability value, M)	1	snoR14 (0.380)	U61 (0.154)	snoR23 (0.044)	*ADP* (0.041)	snoR14 (0.119)	snoR23 (0.04)
	2	*ADP* (0.554)	snoR14(0.184)	snoR14 (0.061)	snoR14 (0.078)	U61 (0.141)	snoR14 (0.045)
	3	snoR23 (0.641)	miR168 (0.223)	*ADP* (0.063)	snoR23 (0.081)	snoR23 (0.151)	*ADP* (0.066)
	4	miR168 (0.708)	*ADP* (0.309)	U61 (0.102)	miR168 (0.104)	miR168 (0.200)	miR168 (0.238)
	5	U61 (0.765)	snoR23 (0.481)	miR168 (0.185)	U61 (0.106)	*ADP* (0.233)	U61 (0.305)
	6	U18 (0.801)	*ACT* (0.567)	*α-TUB* (0.242)	miR159 (0.112)	miR159 (0.259)	*GAPDH* (0.323)
	7	*ACT* (0.883)	*α-TUB* (0.587)	U18 (0.254)	*ACT* (0.129)	U18 (0.269)	*ACT* (0.325)
	8	α-TUB (0.976)	*GAPDH* (0.899)	*ACT* (0.291)	*GAPDH* (0.245)	*GAPDH* (0.294)	miR159 (0.353)
	9	*GAPDH* (1.88)	U18 (1.244)	miR159 (0.346)	U18 (0.260)	*α-TUB* (0.311)	U18 (0.455)
	10	miR159 (2.205)	miR159 (2.165)	*GAPDH* (0.435)	*α-TUB* (0.326)	*ACT* (0.380)	*α-TUB* (0.657)
BestKeeper (SD)	1	*ADP* (0.573)	snoR23 (0.39)	*ADP* (0.27)	snoR14 (0.075)	miR168 (0.155)	miR168 (0.518)
	2	snoR14 (0.772)	*ADP* (0.45)	snoR14 (0.28)	snoR23 (0.111)	U61 (0.2)	snoR14 (0.813)
	3	snoR23 (0.816)	snoR14 (0.61)	snoR23 (0.313)	*ADP* (0.177)	snoR23 (0.222)	snoR23 (0.871)
	4	miR168 (0.972)	miR168 (0.7)	U61 (0.328)	U61 (0.184)	snoR14 (0.23)	*ADP* (0.935)
	5	*GAPDH* (1.074)	U61 (0.82)	miR168 (0.333)	miR168 (0.185)	*ADP* (0.259)	*GAPDH* (1.35)
	6	*ACT* (1.094)	*GAPDH* (0.89)	*GAPDH* (0.4)	*GAPDH* (0.193)	U18 (0.273)	U61 (1.48)
	7	U61 (1.377)	*ACT* (0.93)	U18 (0.46)	miR159 (0.222)	*GAPDH* (0.298)	*α-TUB* (1.64)
	8	*α-TUB* (1.557)	*α-TUB* (1)	*α-TUB* (0.51)	*ACT* (0.307)	*α-TUB* (0.31)	*ACT* (1.745)
	9	U18 (1.913)	U18 (1.41)	*ACT* (0.56)	U18 (0.439)	miR159 (0.36)	U18 (1.75)
	10	miR159 (3.046)	miR159 (2.73)	miR159 (0.752)	*α-TUB* (0.563)	*ACT* (0.449)	miR159 (1.8)

### Gene Expression Stability and Ranking of Candidate Reference Genes by Bestkeeper analysis

**Fig 2:**
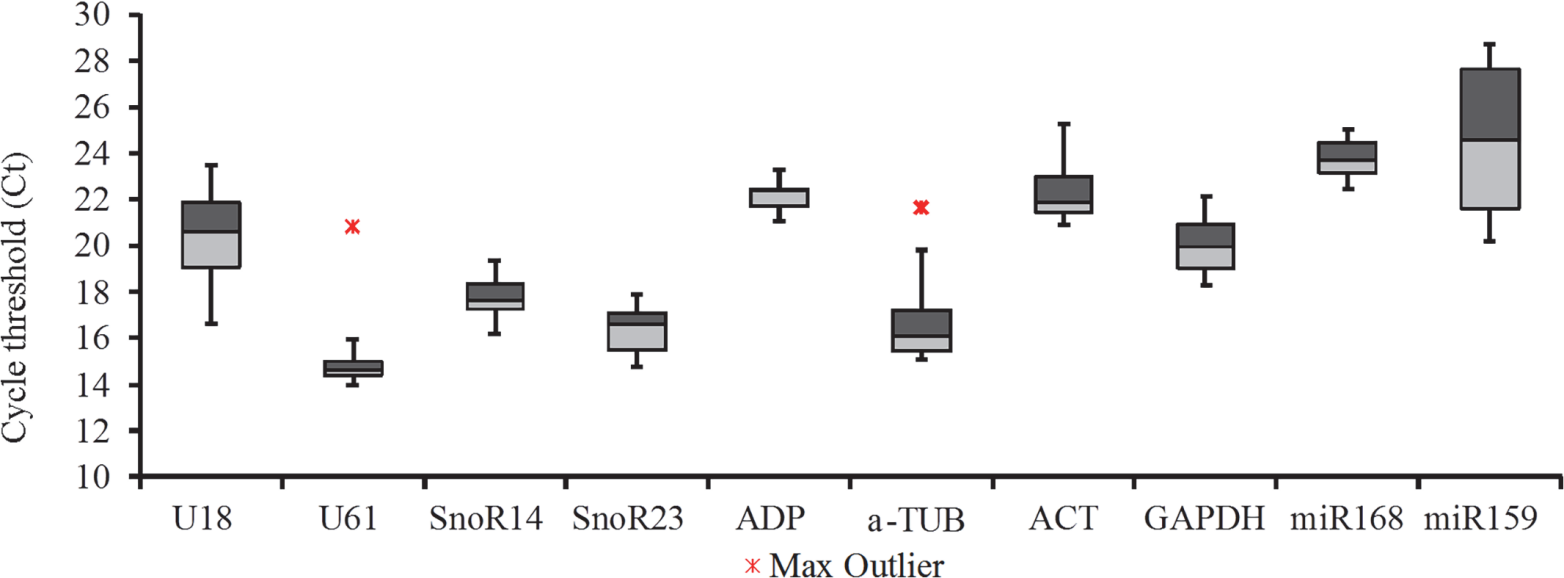
Gene expression of ten candidate internal controls in all samples. Data points are cycle threshold (Ct) values for each candidate reference gene in all samples. Boxes indicate the 25^th^ (light shading) and 75^th^ (dark shading) percentiles, the line indicates the median and whiskers depict the maximum and minimum values.

Expression of the 10 candidate reference genes was measured and Ct values are shown in Fig. 2. Average Ct values for the 10 candidates among all experimental samples varied between 14.98 and 24.35 (Fig. 2); in which higher and lower abundance of mRNA is represented by lower and higher Ct values, respectively. It should be noted that the Ct values obtained for the most stable genes (∼ 20) make them suitable for the normalization of a wide range of experimental targets, but they would be less suitable for certain targets with very high or low expression.

The Bestkeeper analysis suggested that *ADP* (0.573) was the most stable reference gene followed by snoR14 (0.772) and snoR23 (0.816), while miR159 (3.046) was the least stable across all samples (Table 2). SnoR14, snoR23 and *ADP* also performed well in drought, boron and fungal treatment experiments (Table 2). Under salt and nitrate treatments miR168 was the most stable reference gene. miR159 was the least stable candidate under drought, boron and nitrate treatments, while *α-TUB* and *ACT* were the least stable reference genes under fungal and salt treatments, respectively.

**Fig 3:**
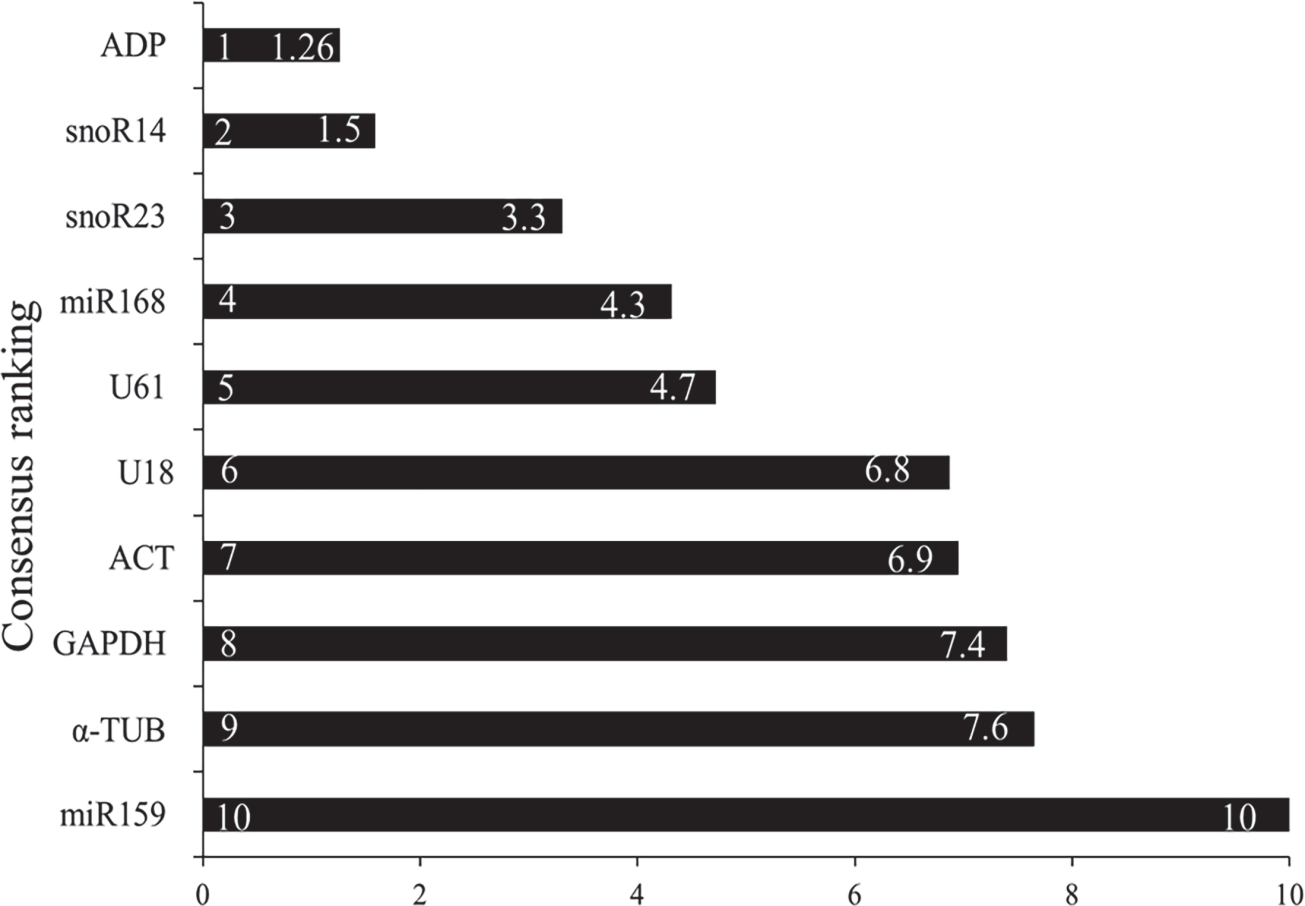
Consensus ranking of ten reference genes. Each candidate gene was assessed in the composite of all samples (n = 44). For each candidate, the consensus ranking was calculated as geometric mean of all rankings suggested by three algorithms; geNorm, Norm Finder and Bestkeeper.

Consensus ranking performance of each candidate reference gene in all samples (*n* = 44) was evaluated by the three algorithms (Fig. 3). For each candidate, the consensus ranking was calculated as geometric mean of three rankings suggested by three algorithms; geNorm, Norm Finder and Bestkeeper.

### The Optimal Number of Reference Genes

**Fig 4:**
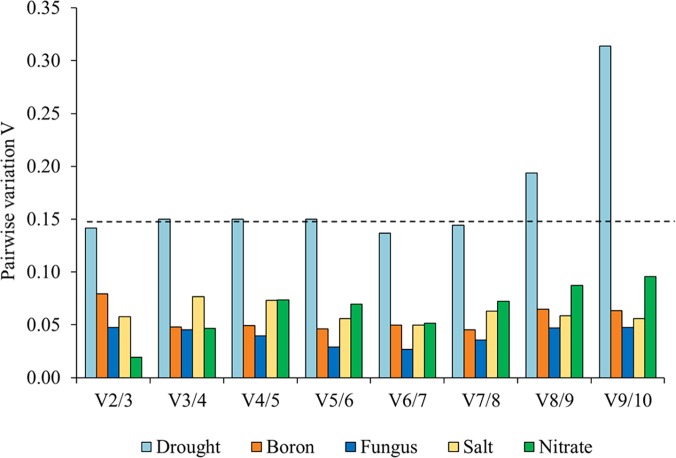
Determination of the optimal number of reference genes for normalization of qPCR data in each experimental condition. If the V (variation) value is below 0.15, the addition of a further reference gene does not result in any improvement of normalization.

The optimal number of reference genes under individual treatment was determined by geNorm analysis (Fig. 4). Under boron, fungal, salt and nitrate treatments, the V_2/3_ values were less than 0.15 (Fig. 4). This analysis suggested that, apart from the two most stable reference genes found in the respective treatment conditions, the addition of a third, reference genes did not improve the normalization so that the use of the two most stable reference genes would be sufficient for normalization. Under drought treatment, V_2/3_-V_7/8_ was ≤ 0.15, while V_8/9_ and V_9/10_ was > 0.15 (Fig. 4). In such a situation, the use of between two and seven reference genes resulted in reliable normalization. It is worth noting that the V value is only a guideline for the determination of an optimal number of reference genes and thus should not be considered as an exact cut-off point.

### Validation of Stable Reference Genes for mRNA and miRNA Expression Under Various Stress Conditions

**Fig 5:**
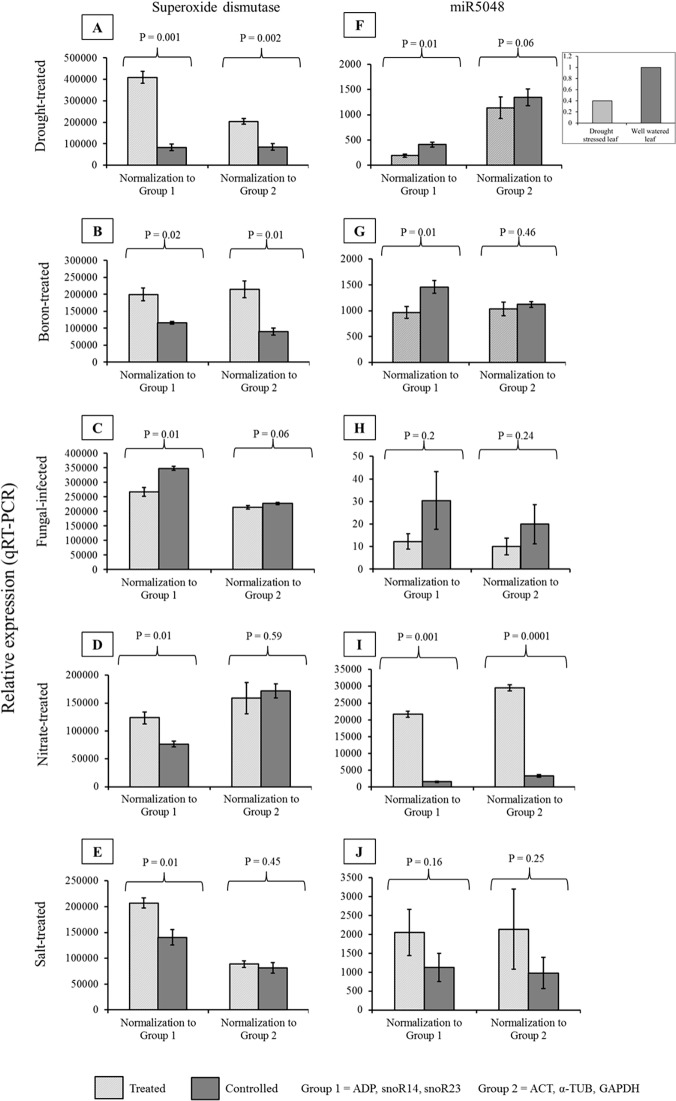
Validation of putatively stable reference genes. Comparison of relative expression of (A, B, C, D, E) *SUPEROXIDE DISMUTASE* (AK363344.1) and (F, G, H, I, J) miR5048 (MIMAT0020544) in drought-treated, boron treated, fungal infected, nitrate treated and salt treated samples and their respective controls by qPCR when normalized to group1 (a combined group of three stable reference genes; snoR14, *ADP*, snoR23) and group 2 (a combined group commonly used housekeeping genes; *ACT*, *α-TUB*, *GAPDH*). The error bars indicate the standard deviation of the mean. Statistical analysis by t-test. (Inset in 5F) The relative expression of miR5048 from deep sequencing of drought-stressed and well-watered barley leaves. Reads per million (RPM) of 1 was the highest number of counts.

To validate the stability of reference genes, we used two groups for normalization. Group 1 denotes the three putatively stable reference genes, *ADP*, snoR14 and snoR23, suggested by the consensus ranking, and group 2 denotes the three commonly used HKGs, *ACT*, *α-TUB* and *GAPDH*. For each experimental condition, the expression of *SUPEROXIDE DISMUTASE* was normalized to group 1 and group 2 in the treated and control samples (Fig. 5). The formation of reactive oxygen species (ROS) in plants is triggered by various environmental stresses, such as drought, nutrient deficiency, nutrient toxicity, salinity and pathogen attack [46, 47]. *SUPEROXIDE DISMUTASE* is one of the first line of defence antioxidant enzymes. Though its expression is not broadly reported for each particular stress condition, it is well-known to be up-regulated under drought stress in plant species including barley [48–55]. As expected, normalized expression of *SUPEROXIDE DISMUTASE* to group 1 and group 2 resulted in lower expression under well-watered (control) than under drought-treated samples (Fig. 5A). However, the expression difference between the treated and control samples was greater when normalized with group 1 candidates (Fig. 5A). In the boron treatment experiment, both normalizer groups showed similar results where *SUPEROXIDE DISMUTASE* was significantly up-regulated in the treated samples (Fig. 5B). In the fungal infected samples the expression level of *SUPEROXIDE DISMUTASE* was reduced compared to the control samples when normalized to group 1. However, when normalized to group 2 the expression level of *SUPEROXIDE DISMUTASE* was not significantly different (Fig. 5C). In the nitrate and salt treatment samples, group 1 normalization resulted in significantly higher *SUPEROXIDE DISMUTASE* expression than in the control samples (Fig. 5D & E). However, in both of these experimental conditions, group 2 normalization did not show any significant difference of *SUPEROXIDE DISMUTASE* expression between the treated and control samples (Fig. 5D & E).

We also quantified the relative expression of a barley miRNA, miR5048, normalizing with the same groups of candidates. The normalization against group 1 reference genes resulted in down-regulation of miR5048 under drought treatment compared with the control conditions (Fig. 5F) which was consistent with our pre-existing deep sequencing data (see inset in Fig. 5F) [45]. In contrast, normalizing the expression level of miR5048 to group 2 candidates resulted in comparatively higher expression of miR5048 with no significant difference between the treated and control samples. This result was inconsistent with the normalized expression using group 1 candidate reference genes as well as inconsistent with our pre-existing deep sequencing result (Fig. 5F and inset). These data indicated an increased sensitivity of detection of differential expression using the putatively more stable group 1 candidates.

We also took advantage of the available boron treated, fungal infected, nitrate treated and salt treated samples to compared miR5048 expression in these conditions normalizing the expression level to the two normalizer groups to check for the groups’ comparative sensitivity.

Under the boron treatment, miR5048 expression normalized to group 1 showed that miR5048 was down-regulated in the treated samples compared to the control samples. However, normalization to group 2 did not show any difference between the treated and control samples (Fig. 5G). Under the fungal infection, miR5048 expression was not abundant and there was no significant difference in expression with treatment detected using either normalizing group of reference genes (Fig. 5H). Under the nitrate treatment, upon normalization to both normalizer groups, miR5048 was significantly up-regulated compared to the control samples (Fig. 5I). The salt treatment did not result in a significant up-regulation of miR5048 expression with either normalizing group (Fig. 5J).

## Discussion

Normalization is an important requirement for the study of gene expression by qPCR. Random selection of reference genes, which may be influenced by experimental treatments, could cause the misinterpretation of results [1, 11]. An appropriate reference gene should have an invariant level of expression regardless of experimental conditions; however, such genes may be hard to find as plant gene expression is affected by environmental conditions [56–59]. It is clear from recent studies that internal reference genes for qPCR assays should be specifically selected for the experimental treatment of interest in both plants and animals [32, 33, 60, 61].

In our study, we evaluated the expression stabilities of ten candidate reference genes, *ACT*, *α-TUB*, *GAPDH*, *ADP*, snoR14, snoR23, U61, U18, miR159 and miR168, in samples from barley plants grown in five stressed conditions. There is no universally accepted method for reference gene selection and stability analysis. We hypothesized that small, non-coding RNAs could be more suitable reference genes for qPCR normalization of miRNAs when compared with commonly used protein-coding reference genes using three popular algorithms in different computer programs such as geNorm, NormFinder and BestKeeper. Using these different analytical algorithms the selected snoRNAs (snoR14 and snoR23) consistently ranked amongst the best and most suitable reference genes, whether individually or in combination, with only *ADP* amongst the more commonly used protein-coding reference genes ranked more highly than other snoRNAs in a composite of all samples by two of the three algorithms.

It is necessary to validate reference genes under each set of experimental conditions [62]. geNorm, NormFinder and BestKeeper analyses were performed separately for each experimental treatment and indicated that two of the candidate snoRNA reference genes, snoR14 and snoR23, were steadily stable in an experiment specific manner. snoR14, in particular and in combination with at least one other reference gene, had potential as a universal reference gene in all the studied sample conditions. Amongst protein-coding genes, *ADP* was indicated as a good potential internal control under drought treatment. This result is consistent with a previous study in barley [9]. Under salt stress, the snoRNA U61 showed stable expression and consistently ranked in the top two potential reference genes by three algorithms. U61 also ranked as the fourth most stable reference gene under boron treatment, miRNAs were also proposed as reference genes for qPCR data normalization of both miRNA and protein-coding genes in an experiment in soybean [1]. The miRNAs, miR168 and miR159, behaved very differently from each other as reference genes in the experiments reported here, underlining the importance of empirical selection and knowledge of the individual gene’s differential expression with treatment. miR168 was selected as a candidate because it was reportedly not significantly affected by drought in *Arabidopsis* [63] and rice [64]. Genome-wide deep sequencing of peach also indicated that there was no change in the expression of miR168 in drought-stressed leaf and root tissues [65]. Our results confirmed the comparatively consistent stability of miR168 as a reference gene under each of the experimental conditions, where it ranked in the top five using the three algorithms.

geNorm and BestKeeper specify a gene expression stability threshold value above which a candidate reference gene should be considered as an unreliable internal control. The threshold value is M = 1.5 in geNorm, a value exceeded by miR159 and *GAPDH* in a composite of all samples. The reliability threshold of SD = 1 in BestKeeper was also a value exceeded by miR159 and *GAPDH*, as well as U61, U18, and *α-TUB* in a composite of all samples.

Though the selection of miR159 was based on our previous miRNA expression studies in drought-treated barley ‘Golden Promise’ (data not shown), miR159 was ranked as the least stable candidate in drought-treated samples by three algorithms. An explanation of this inconsistency under drought treatment may reside in the use of different varieties, developmental stages and the method of quantification which may lead to changes in miRNA expression as well as impact on gene regulation. Additionally, miR159 was ranked as the least stable candidate under boron and nitrate treatments by BestKeeper. miR159 expression also varied in roots under drought stress in peach [65] and showed variable patterns in rice between young and old leaves [66]. Hence we do not consider miR159 a suitable reference gene.

Conventionally used HKGs, those involved in basic cellular mechanisms, have been used extensively for quantification of transcript expression. However, it has also been reported that their expression levels are not completely independent of the exogenous conditions [9, 13–16]. In our study, we found that some of the commonly used housekeeping genes such as *ACT*, *α-TUB* and *GAPDH* were not the most suitable HKGs under specific experimental conditions as in most cases they were ranked lower than the other candidates (Table 2). Additionally, in BestKeeper analysis, the reliability threshold SD = 1 was exceeded by *ACT*, *GAPDH* and *α-TUB* when used as HKGs for nitrate-treated samples demonstrating the importance of selecting reference genes specific to the experimental treatments. We found that the non-coding RNAs generally outperformed *ACT*, *GAPDH* and *α-TUB*. These results suggest that the common HKGs for qPCR data normalization should be used carefully, demanding a thorough evaluation for every experimental set of samples before use. Additionally, comparison of different algorithms for reference genes selection may facilitate a reliable evaluation of stably expressed genes as well as precise data normalization.

In our analysis of the stability of candidate reference genes, we validated their performance by normalizing the relative expression of an mRNA and an miRNA in barley under five experimental conditions. For evaluating mRNA expression, we selected *SUPEROXIDE DISMUTASE* whose enzymatic product rapidly scavenges reactive oxygen species in plants under various environmental stresses [46, 47, 67–70]. Increased expression of *SUPEROXIDE DISMUTASE* has been identified under drought stress in many plant species including cotton [48], pea [49], *Coffea* [50], common bean [51], rice [52], *Populus* [53] and wheat [54, 55]. To validate the performance of stable candidate internal controls for expression data normalization, we used the combination of *ADP*, snoR14 and snoR23, the top three stable reference genes suggested by the consensus ranking. In comparison to this normalization to these putatively stable candidates, we also considered the combination of relatively unstable candidates *ACT*, *α-TUB*, *GAPDH* suggested by the consensus ranking. With both sets of normalizers, *SUPEROXIDE DISMUTASE* expression increased in the drought treatment but the difference of expression between drought and well-watered samples was greater when the stable group of normalizers was used. This increased sensitivity of detection of differential expression was also evident in the fungal infection, low and high nitrate and salt stress experimental samples using the putatively stable group of reference genes. This demonstrated that the choice of appropriate and stable reference genes does increase the sensitivity of experimental assays in qPCR and validated our comparative ranking of the candidates.

To evaluate the putatively stable candidates for normalizing miRNA expression, we selected miR5048 for comparison with our pre-existing deep sequencing data for expression of this miRNA in the barley variety ‘Golden Promise’ under drought and well-watered conditions. Deep sequencing is well-recognised to reflect RNA presence and quantity from a genome at a given stage [71] and should correlate with the expression level identified through qPCR [72]. However, it should be noted that different methods of library construction including choice of adapters and even barcodes could generate significant bias during RNA sequencing profiling of miRNAs [73, 74]. Using the same set of putatively more stable reference genes for miR5048 expression data normalization, miR5048 was down-regulated under drought treatment consistent with the deep sequencing result. However, normalization with the putatively unstable, commonly used *ACT*, *α-TUB* and *GAPDH* failed to detect statistically significant down-regulation of miR5048 under drought stress, conflicting with our deep sequencing result. Thus, the validation of candidate reference genes for normalizing miRNA relative expression profiles increased our confidence in the analysis of stability of *ADP*, snoR14 and snoR23 in drought-stressed barley and highlighted how the use of inappropriate reference genes might lead to erroneous results.

Apart from the drought treatment, as there is lack of reference for miR5048 expression, we could not compare our validation results under individual experimental conditions. Nonetheless, for miR5048 as well as for *SUPEROXIDE DISMUTASE*, there was an increase in the sensitivity of detection of differential expression for boron, fungal and salt treatments when our higher-ranked stable candidates were used, once again validating their superiority.

## Conclusion

We evaluated ten candidate reference genes in different tissues, genotypes and experimental treatments for their ability to normalize miRNA qPCR data. The expression stability of these candidate genes was evaluated across a set of 44 samples using the computer programs geNorm, NormFinder and BestKeeper. We found that two putative snoRNAs, snoR14 and snoR23, outperformed the generally used HKGs in barley. To our knowledge, this work is the first stability evaluation of a set of commonly used and novel putative reference genes for qPCR in barley under different experimental conditions. This study suggests our set of evaluated candidates *ADP*, snoR14 and snoR23 as potential reference genes in barley. Additionally, this work proposes a list of potential candidate reference genes under five, different experimental conditions. We recommend to use a combination of *ADP*, snoR14 and snoR23 as potential reference genes for miRNA and mRNA qPCR data normalization under the experimental conditions mentioned in this study. As the arbitrary selection of internal controls, or the use pre-identified reference genes, may yield inaccurate results, reference gene selection needs to be optimized for individual assays counting a number of candidate reference genes for evaluation. We also recommend the use of multiple reference genes for more reliable and valid normalization of gene expression in barley.

## Supporting Information

S1 Fig Melt curves of the ten candidate internal controls. (TIF)

S2 Fig Agarose gel (3%) electrophoresis showing amplification of the qPCR product of expected size for each candidate internal controls tested in this study. (TIF)

S3 Fig A polymerase chain reaction (PCR) using the mixture of RNA samples as no template controls for each primer sets. (TIF)

S4 Fig Standard curves of the ten candidate internal controls. (TIF)

S5 Fig Melt curve and standard curve of *SUPEROXIDE DISMUTASE*. (TIF)

S6 Fig Melt curve and standard curve of miR5048. (TIF)

S1 Table Primers used for sequencing individual candidate reference genes. (DOCX)

S2 Table Primers used for qPCR of barley *Superoxide dismutase* and miR5048. (DOCX)
