# Correction: Identification of Reference Genes for Quantitative Expression Analysis of MicroRNAs and mRNAs in Barley under Various Stress Conditions

**DOI:** 10.1371/journal.pone.0126167

**Published:** 2015-05-06

**Authors:** The PLOS ONE Staff

The Abstract of the published article is incorrect. Please view the correct Abstract here:

For accurate and reliable gene expression analysis using quantitative real-time reverse transcription PCR (qPCR), the selection of appropriate reference genes as an internal control for normalization is crucial. We hypothesized that non-coding, small nucleolar RNAs (snoRNAs) would be stably expressed in different barley varieties and under different experimental treatments, in different tissues and at different developmental stages of plant growth and therefore might prove to be suitable reference genes for expression analysis of both microRNAs (miRNAs) and mRNAs. In this study, we examined the expression stability of ten candidate reference genes in six barley genotypes under five experimental stresses, drought, fungal infection, boron toxicity, nutrient deficiency and salinity. We compared four commonly used housekeeping genes; Actin (*ACT*), alpha-Tubulin (*α-TUB*), Glycolytic glyceraldehyde-3-phosphate dehydrogenase (*GAPDH*), ADP-ribosylation factor 1-like protein (*ADP*), four snoRNAs; (U18, U61, snoR14 and snoR23) and two microRNAs (miR168, miR159) as candidate reference genes. We found that *ADP*, snoR14 and snoR23 were ranked as the best of these candidates across diverse samples. Additionally, we found that miR168 was a suitable reference gene for expression analysis in barley. Finally, we validated the performance of our stable and unstable candidate reference genes for both mRNA and miRNA qPCR data normalization under different stress conditions and demonstrated the superiority of the stable candidates. Our data demonstrate the suitability of barley snoRNAs and miRNAs as potential reference genes for miRNA and mRNA qPCR data normalization under different stress treatments.
