# Supplementary figures and images for: Identification of Reference Genes for Quantitative Expression Analysis of MicroRNAs and mRNAs in Barley under Various Stress Conditions
Source: PLoS One. 2015 Mar 20;10(3):e0118503. doi: 10.1371/journal.pone.0118503 (PMC4368757; doi:10.1371/journal.pone.0118503)

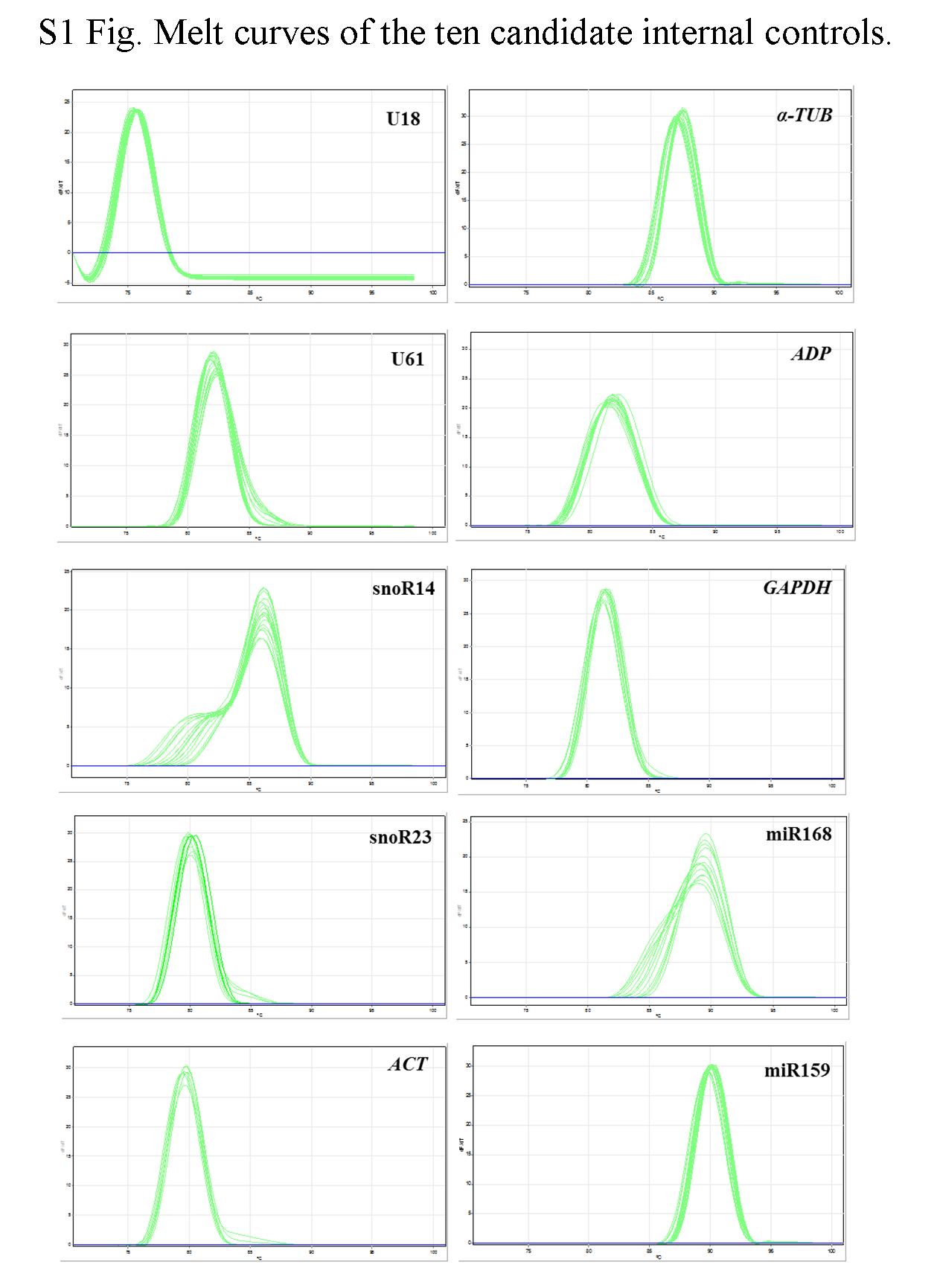

Supplement: S1 Fig — (TIF) [file pone.0118503.s001.tif]

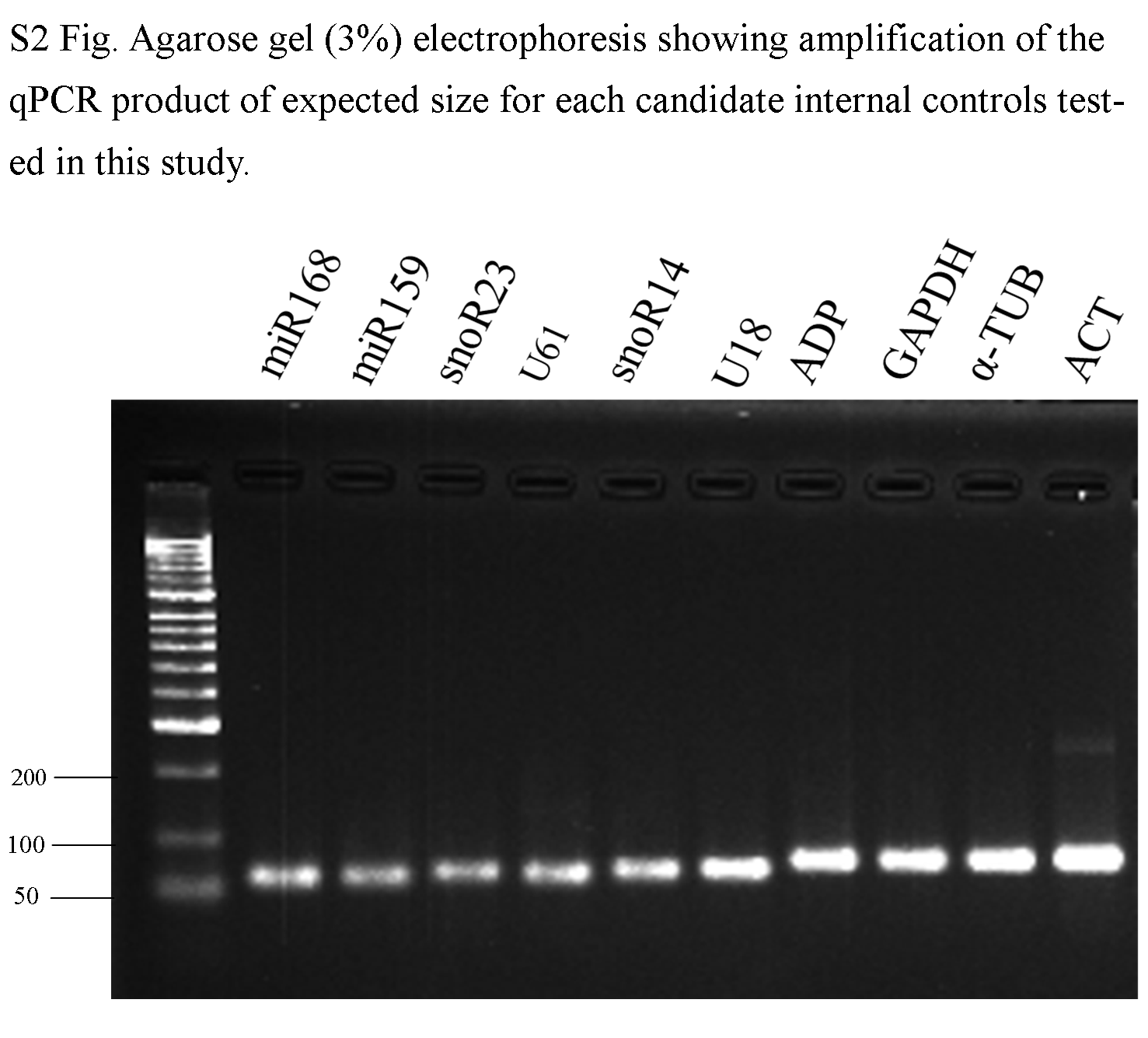

Supplement: S2 Fig — (TIF) [file pone.0118503.s002.tif]

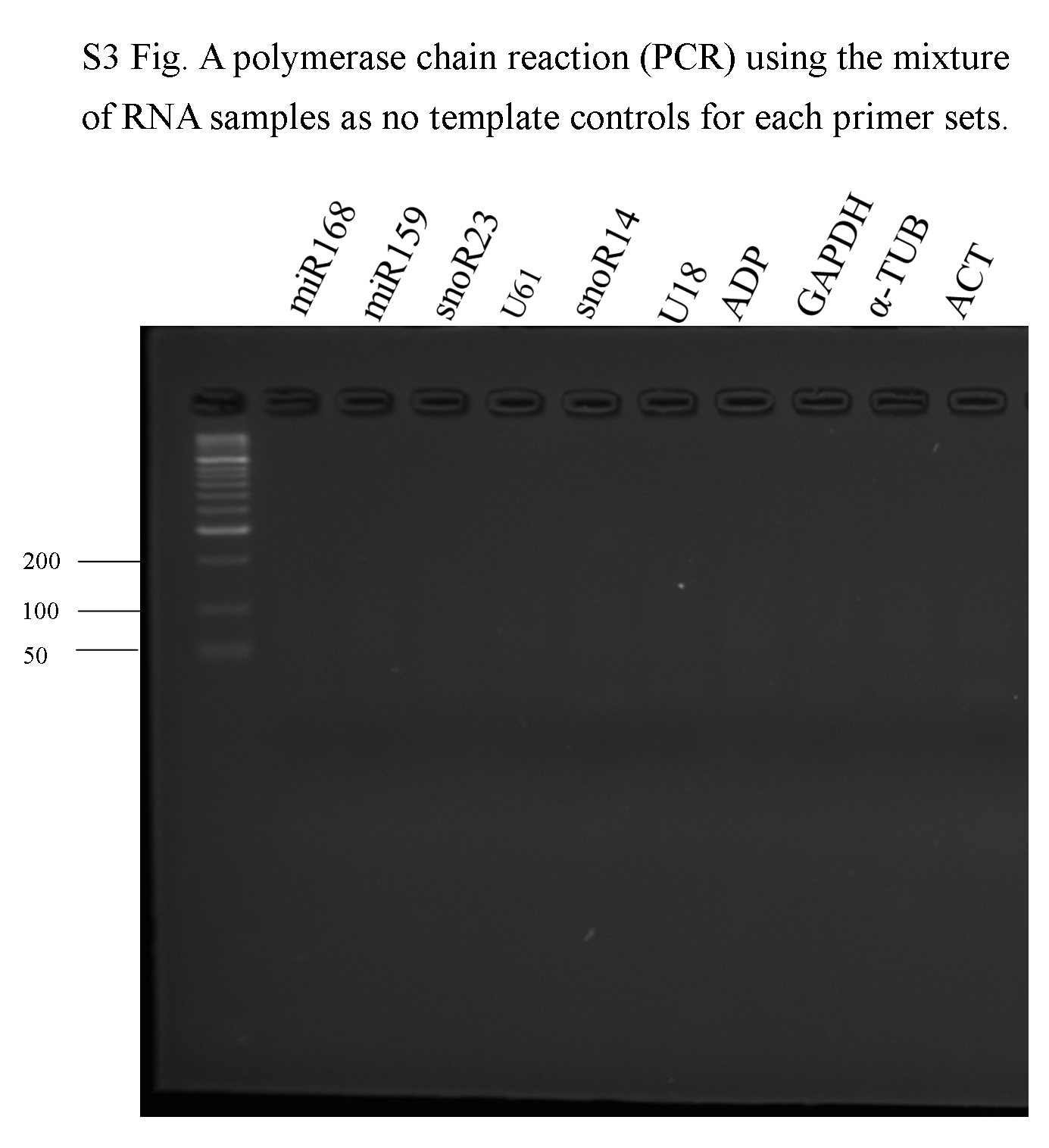

Supplement: S3 Fig — (TIF) [file pone.0118503.s003.tif]

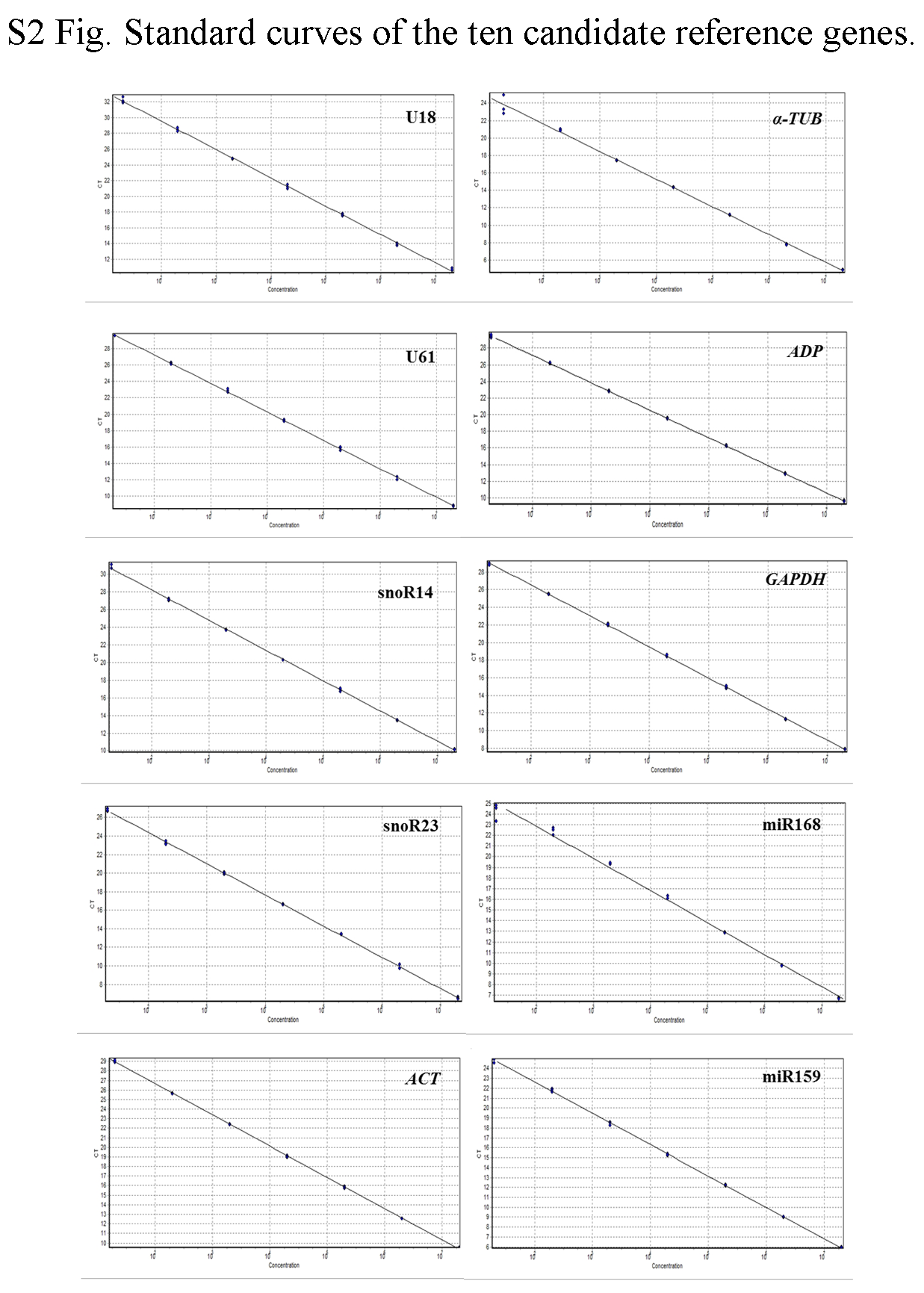

Supplement: S4 Fig — (TIF) [file pone.0118503.s004.tif]

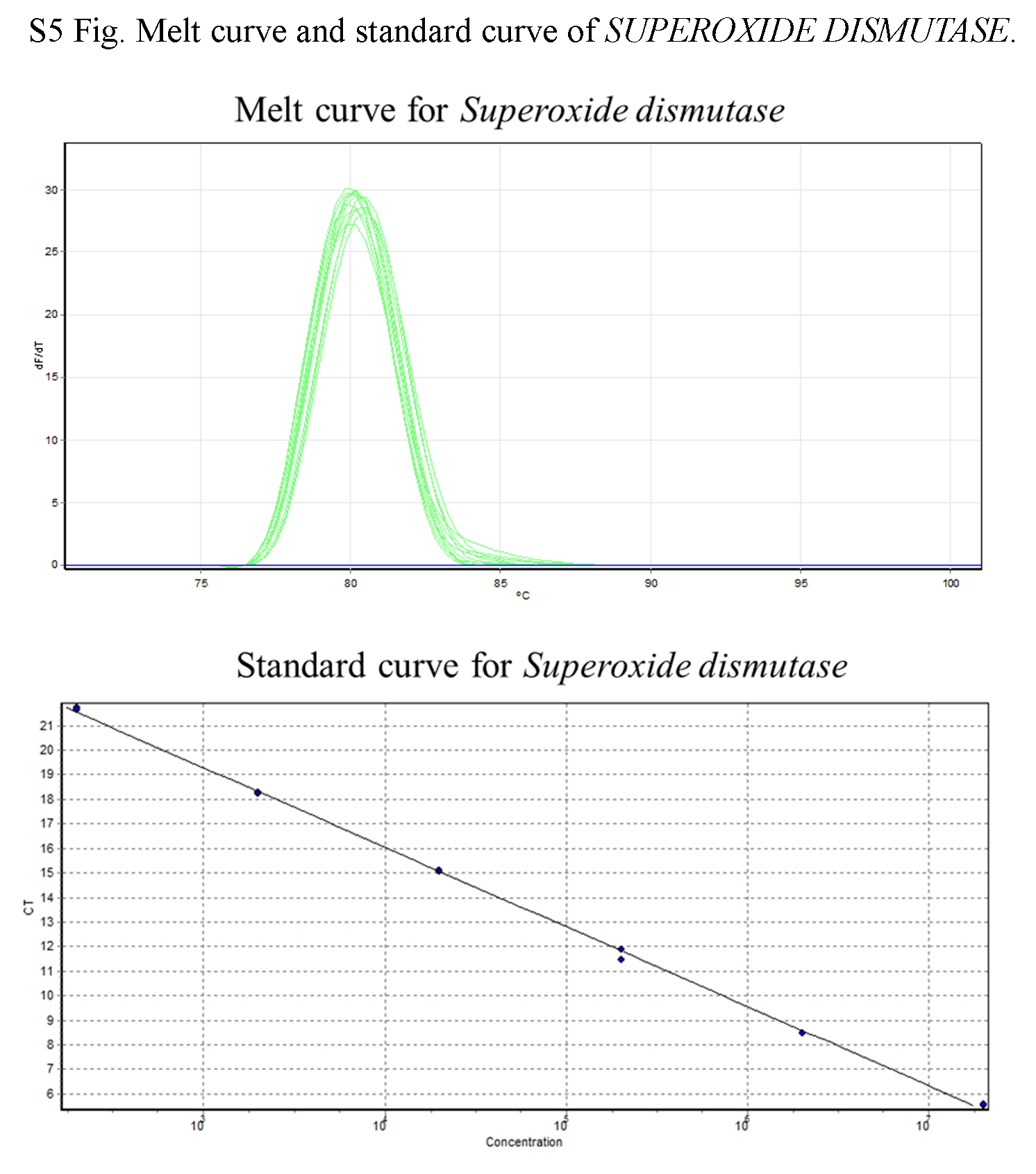

Supplement: S5 Fig — (TIF) [file pone.0118503.s005.tif]

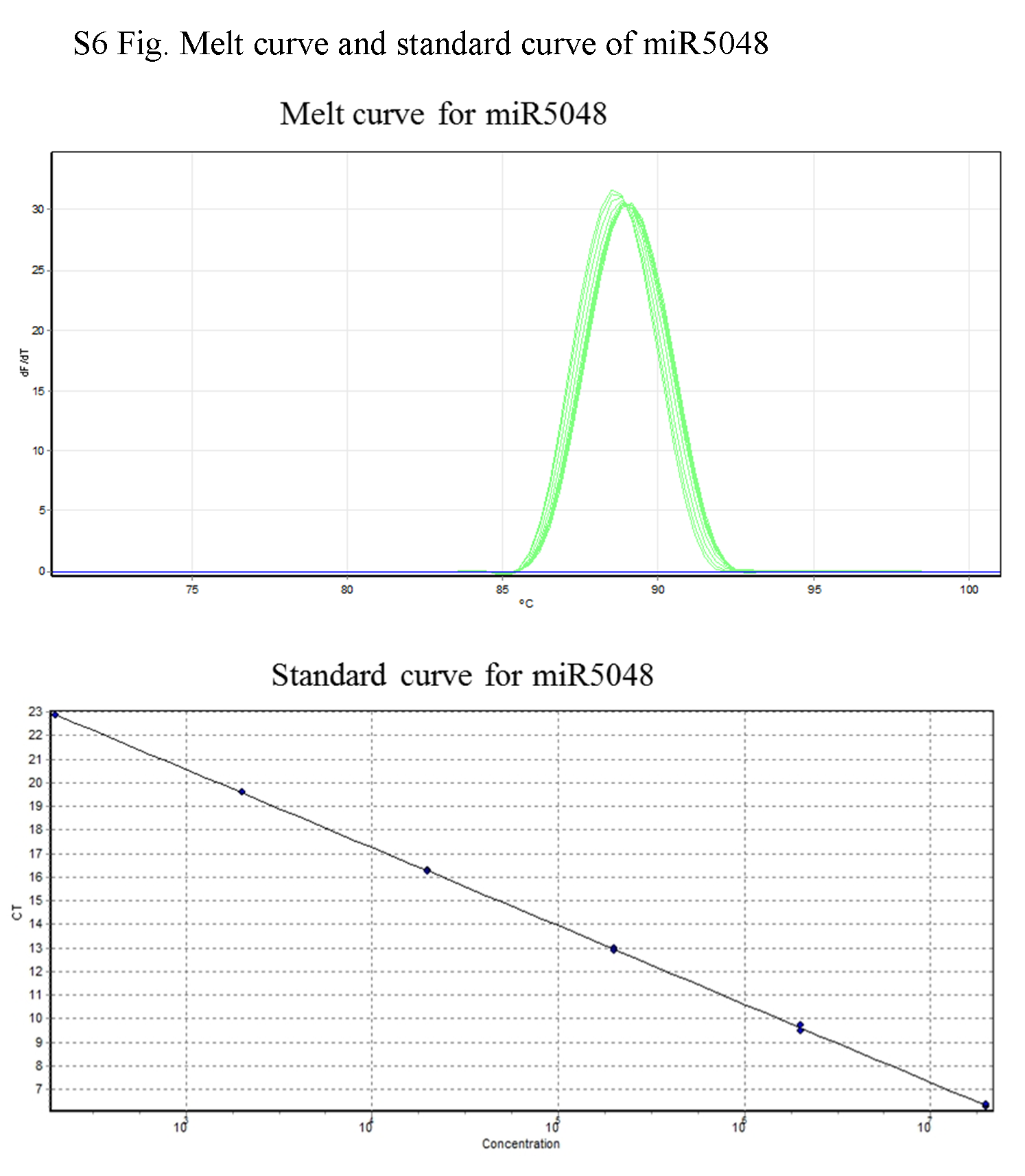

Supplement: S6 Fig — (TIF) [file pone.0118503.s006.tif]
