# Supplementary material for: Identification of Reference Genes for Quantitative Expression Analysis of MicroRNAs and mRNAs in Barley under Various Stress Conditions
Source: PLoS One. 2015 Mar 20;10(3):e0118503. doi: 10.1371/journal.pone.0118503 (PMC4368757; doi:10.1371/journal.pone.0118503)
Supplement: S1 Table — (DOCX) [file pone.0118503.s007.docx]

**S1 Table.** Primers used for sequencing individual candidate reference genes.

| **Primer name** | **Primer sequence (5’–3’)** |
| --- | --- |
| M13r+ SPACER + Hvu-miR168 Rev | CAGGAAACAGCTATGACCATGGATCGATCGAGATCGATCGAGAGCTGGGTCCGAGGT |
| M13r+ SPACER + Hvu-miR159 Rev | CAGGAAACAGCTATGACCATGGATCGATCGAGATCGATCGAGTGCAGGGTCCGAGGT |
| M13r+ SPACER + Hvu-ACT Rev | CAGGAAACAGCTATGACCATGGATCGATCGAGATCGATCGACACTGAGCACGATGTTTCC |
| M13r+ SPACER + Hvu-ADP Rev | CAGGAAACAGCTATGACCATGGATCGATCGAGATCGATCGAGAGACATCCAGCATCATTCATTCC |
| M13r+ SPACER + Hvu-GAPDH Rev | CAGGAAACAGCTATGACCATGGATCGATCGAGATCGATCGACACATTTATTCCCATAGACAAAGG |
| M13r+ SPACER + Hvu-α-TUB Rev | CAGGAAACAGCTATGACCATGGATCGATCGAGATCGATCGACGGCGGCAGATGTCATAGATG |
| M13r+ SPACER + Hvu-snoR14 Rev | CAGGAAACAGCTATGACCATGGATCGATCGAGATCGATCGAGTCGGGATGTATGCGTGTC |
| M13r+ SPACER + Hvu-U61 Rev | CAGGAAACAGCTATGACCATGGATCGATCGAGATCGATCGAACTTCTTAGAGGGTTGTGTTAC |
| M13r+ SPACER + Hvu-U18 Rev | CAGGAAACAGCTATGACCATGGATCGATCGAGATCGATCGAAGAAGTTTATTAAGGATGGTTATC |
| M13r+ SPACER + Hvu-snoR23 R | CAGGAAACAGCTATGACCATGGATCGATCGAGATCGATCGACTCAGTGGAAAGAGAAGTCG |
| M13r+ SPACER + Hvu-miR5048 Rev | CAGGAAACAGCTATGACCATGGATCGATCGAGATCGATCGAGTGCAGGGAGGGAGGT |
| M13 reverse* | CAGGAAACAGCTATGACCATG |
| SPACER# | GATCGATCGAGATCGATCGA |

Note: Underlined sequence is a spacer sequence used for getting a complete sequence of each amplicon, and M13 reverse (M13r) primer was used to sequence all the amplicons.
