# Supplementary material for: Identification of Reference Genes for Quantitative Expression Analysis of MicroRNAs and mRNAs in Barley under Various Stress Conditions
Source: PLoS One. 2015 Mar 20;10(3):e0118503. doi: 10.1371/journal.pone.0118503 (PMC4368757; doi:10.1371/journal.pone.0118503)
Supplement: S2 Table — (DOCX) [file pone.0118503.s008.docx]

**S2 Table.** Primers used for qPCR of barley *Superoxide dismutase* and miR5048.

| Primer name | Primer sequence (5’–3’) | Accession number | Amplicon (bp) | PCR efficiency | Regression coefficient (R^2^) |
| --- | --- | --- | --- | --- | --- |
| Hvu-*Superoxide dismutase* forward | CTTGAAGGACACCGACTTGC | AK363344.1 | 141 | 1.03 | 0.999 |
| Hvu-*Superoxide dismutase* reverse | CTCAAAAAGCCAAATGACAGTG |  |  |  |  |
| Hvu-miR5048 forward | CGTCTTCGGTATTTGCAGGTTTTA | MIMAT0020544 | 65 | 1.0 | 0.999 |
| Hvu-miR5048 reverse | GTGCAGGGAGGGAGGT |  |  |  |  |
| Hvu-miR5048 stem-loop RT primer | GTCGTATCCAGTGCAGGGAGGGAGGTATTCGCACTGGATACGACTTAGAC |  |  |  |  |
